# Decoding Post-Transcriptional Networks Governing the Melanoma Extracellular Matrix

**DOI:** 10.3390/cancers18142326

**Published:** 2026-07-18

**Authors:** Elias N. Katsoulieris, Paraskevi Ioannou, Nikolaos A. Afratis

**Affiliations:** 1 Laboratory of Biochemistry, Biotechnology and Molecular Analysis, Department of Agricultural Development, Agri-Food & Management of Natural Resources, National and Kapodistrian University of Athens, 344 00 Evia, Greece; 2 Department of Immunology and Regenerative Biology, Weizmann Institute of Science, Rehovot 7610001, Israel

**Keywords:** melanoma, microRNAs, extracellular matrix, tumor microenvironment, matrix remodeling, metastasis, matrisome, collagen, proteoglycans, extracellular signaling

## Abstract

The extracellular matrix (ECM) is a key determinant of melanoma progression, regulating tumor cell behavior through biochemical and biomechanical cues. MicroRNAs (miRNAs) have emerged as critical post-transcriptional regulators of gene expression and are increasingly recognized as important modulators of ECM remodeling in cancer. This review summarizes current evidence on miRNA-mediated regulation of ECM components and ECM-associated pathways in melanoma (matrix-miRNAs). We examine studies reporting miRNAs that directly target structural ECM molecules such as collagens, laminins, fibronectin, hyaluronan-related enzymes, and proteoglycans, as well as miRNAs that indirectly regulate ECM dynamics through modulation of matrix metalloproteinases, signaling pathways, and transcription factors. The collected evidence indicates that miRNAs form complex regulatory networks that influence tumor proliferation, invasion, metastasis, angiogenesis, immune evasion, and therapy resistance by reshaping the tumor microenvironment. Tumor-suppressive miRNAs generally inhibit ECM remodeling and metastatic behavior, whereas oncogenic miRNAs promote matrix degradation and a pro-invasive microenvironment. Importantly, many miRNAs exert pleiotropic effects by targeting multiple components of interconnected signaling pathways, resulting in context-dependent outcomes during melanoma progression. Overall, miRNA-dependent regulation of the ECM represents a crucial layer of control in melanoma biology. Understanding these regulatory circuits may provide novel opportunities for biomarker development and therapeutic intervention targeting both tumor cells and their surrounding microenvironment.

## Simple Summary

Melanoma is an aggressive skin cancer whose progression is strongly influenced by the extracellular matrix (ECM), a dynamic network of proteins and polysaccharides surrounding tumor cells. Increasing evidence indicates that microRNAs (miRNAs), small non-coding RNAs that regulate gene expression, contribute substantially to melanoma development by controlling ECM composition, remodeling, and signaling. These regulatory interactions influence tumor cell proliferation, invasion, metastasis, immune evasion, and therapeutic resistance. This review summarizes current knowledge regarding miRNAs that directly target ECM molecules or indirectly regulate ECM turnover through transcription factors, signaling pathways, and matrix-remodeling enzymes. We highlight how these post-transcriptional networks shape the melanoma microenvironment and discuss their potential as biomarkers and therapeutic targets. A better understanding of miRNA–ECM interactions may facilitate the development of innovative approaches for melanoma diagnosis, prognosis, and treatment.

## 1. Introduction

The extracellular matrix (ECM) is a complex, dynamic, three-dimensional network of biomolecules that surrounds cells and offers structural support along with vital regulatory signals for tissue function and homeostasis [1,2,3]. Composed primarily of collagens, elastin, laminins, fibronectin, proteoglycans (PGs), glycosaminoglycans (GAGs), and various other glycoproteins, the ECM determines both the biochemical and mechanical properties of tissues [3,4]. Importantly, the ECM’s composition and spatial organization are continually remodeled in response to physiological processes such as development and wound healing or pathological conditions such as fibrosis, inflammation and cancer [4,5,6]. The skin itself is a specialized organ made up of the epidermis, dermis, and hypodermis, which contains dermal adipose tissue and fascia [7]. The epidermis is attached to the dermis by the basement membrane, an ECM-rich structure that links it to the dermal interstitial matrix [8]. The dermis contains two major layers: the papillary dermis, which is thinner and richer in cells, and the reticular dermis, which is thicker and packed with fibrous ECM components. These layers differ in ECM composition, organization and biomechanical properties [8,9].

MicroRNAs (miRNAs) are small, evolutionarily conserved 18–24-nucleotide-long non-coding RNA molecules, first discovered in *C. elegans* over two decades ago [10]. Since their discovery, miRNAs have emerged as prominent post-transcriptional regulators of gene expression, modulating target mRNA levels in response to both cellular homeostasis and environmental stimuli [11,12,13]. Typically, miRNAs interact with complementary sequences within the 3′ untranslated region (3′UTR) of target mRNAs via a 6–7-nucleotide-long 5′ “seed” sequence [14], resulting in translational repression or mRNA degradation via endonucleolytic cleavage, decapping or poly(A) tail deadenylation [11,12,13]. The broad regulatory role of miRNAs is underscored by findings that at least 30% of protein-coding genes are regulated by miRNAs [14] and approximately 60% of human protein-coding genes contain at least one conserved miRNA binding site, alongside numerous non-conserved sites [15]. Given that a single miRNA can potentially regulate hundreds of mRNAs [16], miRNAs exert broad influence across cellular pathways.

In melanoma, miRNAs are critically involved in tumor initiation, progression, metastasis, and therapeutic resistance [17]. Dysregulated miRNA expression can activate oncogenic pathways or suppress tumor-suppressive mechanisms, thereby influencing melanoma cell proliferation, invasion, angiogenesis, and immune evasion [18]. For instance, oncogenic miRNAs such as miR-21 are frequently upregulated, facilitating melanoma progression, whereas tumor-suppressive miRNAs, including miR-34a and miR-211, often exhibit reduced expression. Due to their stability in cells and bodily fluids, miRNAs have emerged as promising biomarkers for melanoma diagnosis, prognosis, and treatment monitoring [19]. Correspondingly, therapeutic strategies targeting the restoration or inhibition of specific miRNAs are actively being explored to enhance melanoma management and combat drug resistance [20].

MicroRNAs that directly target ECM components or functionally regulate ECM-remodeling enzymes, ECM-associated receptors, or ECM-mediated signaling pathways are referred to here as matrix-miRNAs (matrix-miRs). Collectively, these miRNAs participate in regulatory networks that influence ECM architecture and remodeling in health and disease. Many matrix-miRs also engage in bidirectional interactions with the ECM, forming positive or negative feedback loops that further shape the melanoma tumor microenvironment. Matrix-miRs influence ECM component levels and, consequently, ECM-mediated signaling, primarily via integrin-, CD44- and hyaluronan-mediated pathways, which can reciprocally affect the expression of certain matrix-miRs [16] and collectively shape ECM architecture. For instance, MMP-9 signaling can alter methylation of the miR-494 promoter, a syndecan-1-targeting miRNA, thereby reducing its expression and increasing syndecan-1 levels [21,22,23,24]. Additionally, aberrant ECM turnover provides tumorigenic cues that support cancer cell metastasis and involves miRNA-mediated regulation during ECM biosynthesis, modification and degradation [25,26]. Elastin degradation products and proteolytic fragments, including endostatin and angiostatin, exert antiangiogenic effects [27,28,29], while elevated heparanase activity in primary melanomas is linked to increased metastatic risk [30] through basement membrane degradation and activation of tumorigenic pathways [31] involving GEF-H1 and the RhoA oncogene [32]. Notably, miR-1258, which targets heparanase, reduces metastatic potential across various cancers [33,34], and miR-194 negatively regulates the GEF-H1 pathway in melanoma [35]. Artificial miR-30-based shRNAs targeting heparanase have been shown to reduce melanoma metastases in in vivo xenograft models [36].

To facilitate discussion of matrix-miR regulation in melanoma, this review is organized by three major ECM compartments sequentially implicated in tumor progression and metastatic dissemination: the interstitial matrix, the basement membrane, and the cell surface-associated matrix, since the role of MMPs has been mentioned in a previous review [37]. While these structures are functionally interconnected and collectively constitute the tumor microenvironment (TME), they vary distinctly in composition, organization, and biological function. The interstitial matrix, comprising the majority of the dermal TME, is rich in fibrillar collagen (notably type I and III), fibronectin, elastin-associated proteins, matricellular proteins, and proteoglycans, providing the biomechanical scaffold governing cell migration, invasion, angiogenesis, and immune cell infiltration. Key molecules such as EMILINs, tenascin-C, periostin, and versican contribute to matrix remodeling and metastatic niche formation within this compartment. Conversely, the basement membrane, a specialized ECM separating the epidermis from the underlying dermis, consists primarily of collagen IV, laminins, nidogens, and heparan sulfate proteoglycans. Maintenance of basement membrane integrity poses a significant barrier to melanoma invasion, whereas its degradation facilitates vertical growth and metastatic dissemination. Finally, the cell surface-associated matrix—composed of transmembrane receptors and glycocalyx elements, like syndecans, glypicans, CD44, and hyaluronan complexes—mediates cell–ECM interactions and transduces biochemical and mechanical signals vital for regulating melanoma cell behavior. Despite a separate discussion for clarity, these compartments are subject to continuous reciprocal remodeling throughout melanoma progression and should be considered dynamic, interconnected constituents of the extracellular ecosystem.

## 2. MicroRNAs Targeting Interstitial Extracellular Matrix Molecules

The abundance of ECM components, such as collagen, in melanoma growth, progression and metastasis has yielded conflicting results [38,39], making it difficult to determine whether a collagen-rich ECM promotes or impedes melanoma spread. For example, variations in collagen deposition sites—as observed with hyaluronan and proteoglycan link protein 1 (HAPLN1)—may differentially impact melanoma progression: perivascular collagen deposition may act as a barrier to extravasation, whereas peritumoral collagen might enhance tumor cell migration and invasion. miR-134-mediated inhibition of collagen synthesis (by targeting collagen triple helix repeat containing 1, CTHRC1) has been demonstrated to suppress melanoma growth and metastasis in clinical specimens and melanoma cell lines [40,41], with increased CTHRC1 expression correlating with an invasive phenotype in primary melanomas [42], suggesting a collagen dependency influenced by tumor stage or growth signals.

Collagen cross-linking within the TME, much like collagen linearization, plays a crucial role in promoting metastasis [43]. Prolyl hydroxylases (PHs) catalyze collagen crosslinking during collagen synthesis before secretion, while lysyl oxidases (LOXs), lysyl oxidase-like enzymes (LOXLs) and tissue transglutaminase 2 catalyze the cross-link formation between collagen chains and other ECM constituents such as elastin or fibronectin [44]. miR-30a targets LOX and has demonstrated anti-metastatic effects in melanoma cell lines [45,46]. The ECM protein periostin (POSTN) also enhances collagen cross-linking in a LOX-dependent manner [47] and is produced by both melanoma cells and melanoma-associated fibroblasts [48]. POSTN is involved in cell adhesion, collagen fibrillogenesis and EMT [49]. Additionally, POSTN ties skin inflammation to melanoma progression in humans and mice; increased M2 macrophages associated with melanoma correspond to elevated POSTN expression and poor prognosis [50]. PHs are targeted by various miRNAs—including miR-17/20, miR-124 and miR-429 [51,52,53]—all of which act as melanoma invasion suppressors according to independent studies [54,55,56]. Although these miRNAs do not directly affect the modification enzyme, they link miRNA function with metastatic outcomes. The only tumor suppressor miRNA shown to target POSTN in melanoma is miR-214 [57]. Taken together, miRNAs that inhibit the proliferation, migration and invasion of melanoma cells also tend to target collagen cross-linking enzymes or those that regulate them.

Age-related ECM changes represent another predisposing factor for melanoma development. Aging is associated with decreased collagen density and increased collagen fiber linearization, both of which promote cancer cell migration and progression [58,59]. This process is facilitated by matricellular Wnt1-inducible-signaling pathway protein 1 (WISP1) binding to linearized collagen; WISP1 is regulated by miR-134 and miR-1238 in uveal melanoma [60], with both miRNAs acting as tumor suppressors but demonstrating reduced expression in melanoma, possibly explaining the observed collagen linearization. WISP2, an inhibitor of WISP1–collagen interaction, is typically diminished in solid melanomas, which correlates with increased EMT rates and metastasis [61], though miRNA regulation of WISP2 in melanoma remains unexplored.

The aging skin is additionally characterized by reduced expression of fibroblastic HAPLN1, which leads to destabilization of proteoglycans and subsequent downregulation of VE-cadherin junctions between lymphatic endothelial cells, allowing greater metastatic potential for melanoma in lymphatic tissues [62,63]. In contrast, melanoma cells secrete exosomes containing HAPLN1 [64], which serves to promote tumor growth and cell migration via NF-kB and MMP-2-mediated mechanisms [65], while CAFs can also constitute a source for metastasis-inducing HAPLN1 [66]. Accordingly, this case illustrates that a specific ECM component exhibits distinct expression and functional patterns across various tissues, including tumoral tissue, which collectively contribute to the promotion of melanoma metastasis. Recent predictive models indicate that melanoma exosomal HAPLN1 is targeted by hsa-miR-23, which has been demonstrated to inhibit melanoma metastasis primarily through the suppression of autophagy [64]. Notably, miR-21 inhibits TIMP3 and subsequently activates MMPs in in vivo melanoma models [67].

**Figure 1:**
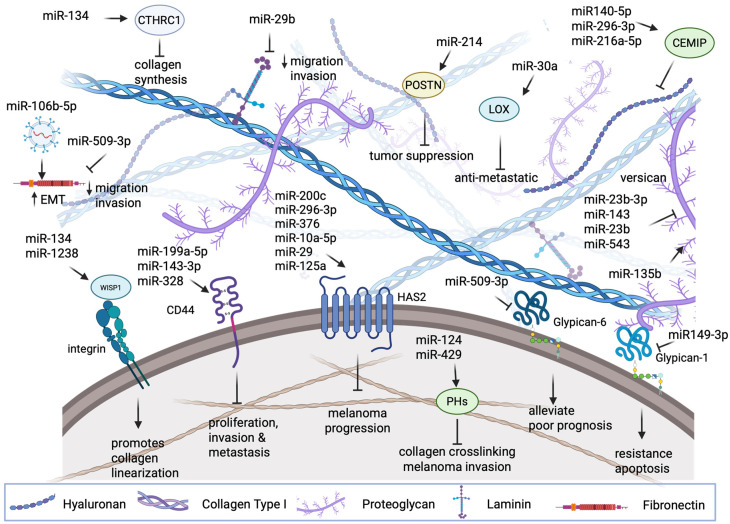
MicroRNAs regulating extracellular matrix components and remodeling enzymes in melanoma progression. Schematic overview of microRNAs implicated in the regulation of ECM constituents and ECM-associated proteins involved in melanoma development and metastasis. Several miRNAs, including miR-134 and miR-29b, suppress collagen deposition and collagen-dependent invasion through the inhibition of CTHRC1 and collagen-associated pathways, while miR-124 and miR-429 target prolyl hydroxylases (PHs), limiting collagen maturation and matrix crosslinking. Likewise, miR-214 and miR-30a inhibit POSTN and LOX, reducing ECM stiffening and the establishment of a prometastatic microenvironment. Regulation of proteoglycans and hyaluronan metabolism by miRNAs, including miR-23b-3p, miR-143, miR-135b, miR-543, miR-509-3p, miR-149-3p, and miR-200c, together with other HAS2-targeting miRNAs, further modulates cell adhesion, migration, and communication with the tumor microenvironment. In addition, miR-140-5p, miR-296-3p, and miR-216a-5p suppress CEMIP, thereby influencing hyaluronan turnover and ECM-dependent melanoma cell motility, whereas miR-199a-5p, miR-143-3p, and miR-328 inhibit CD44, attenuating melanoma cell proliferation, invasion, and metastatic potential. Finally, regulation of WISP1 by miR-134 and miR-1238 influences collagen organization through integrin signaling. Overall, the figure demonstrates that miRNAs do not regulate isolated ECM components but instead coordinate interconnected pathways controlling ECM composition, remodeling, and cell–matrix interactions. This integrated regulatory network shapes the biomechanical and signaling properties of the tumor microenvironment, ultimately determining melanoma invasion, dissemination, and metastatic progression (Table 1) (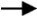 induction, 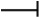 inhibition).

Hyaluronan promotes metastasis through interaction with CD44; miR-199a-5p and miR-143-3p have been shown to target CD44 in malignant melanoma, restricting invasion and metastasis [68,69]. Similarly, cell migration-inducing hyaluronidase 1 (CEMIP), an enzyme responsible for degrading hyaluronan into signaling fragments that promote tumor progression [70], is subject to regulation by miRNAs, such as miR-140-5p [71], miR-296-3p [72], and miR-216a-5p [73]. Independent studies using melanoma models have demonstrated that these miRNAs operate concurrently to either directly or indirectly suppress the expression of MMP-2, MMP-7, and MMP-9 or interfere with glycolytic activity, thereby restricting melanoma growth and invasiveness [74,75,76]. Furthermore, miR-200c, miR-296-3p, miR-376, miR-10a-5p, miR-29 and miR-125a all target isoforms of hyaluronan synthase (HAS) [77,78,79,80,81], regulating hyaluronan biosynthesis while also affecting melanoma growth and aggressiveness through alternate pathways, such as NF-kB and MAPK inhibition, LIN2B (a let-7 suppressor), MMP-2/9 and insulin growth factor receptor (IGFR) expression [76,82,83,84]. These findings underscore that multi-targeting miRNAs modulate matrisome-relevant transcripts, amplifying melanoma’s aggressiveness through multiple tumorigenic pathways. Nevertheless, further investigation is necessary to confirm whether miRNA-mediated melanoma suppression via these alternative mechanisms coincides with concurrent targeting of HAS (Figure 1).

## 3. MicroRNAs Targeting Basement Membrane and Matricellular ECM Molecules

Melanoma cells release exosomal miR-106b-5p, which promotes EMT in nearby tumor cells and melanocytes, partly by increasing fibronectin expression in target cells [85]. When miR-509-3p targets fibronectin, it decreases melanoma cell migration and invasiveness in vitro [41], whereas miR-143 boosts the metastatic potential of liver carcinomas by suppressing fibronectin [86]. Interestingly, miR-143 also inhibits melanoma cell proliferation, EMT and aggressiveness [87], though its direct effect on fibronectin in these processes remains unclear.

Studies beyond melanoma have identified miRNAs targeting tenascin-C (TNC), such as miR-218 and miR-495-3p [88]—both implicated in melanoma progression [89]. However, evidence for miRNAs directly targeting TNC in melanoma mainly comes from prediction analyses, highlighting miR-4443 and miR-3620 as potential regulators [86]. The reported 99% probability that these miRNAs target TNC is related to downstream ECM modulatory effects triggered by NF-kB downregulation, including MMP-9 inhibition [90]. The impact of these two miRNAs, along with other known TNC-regulatory miRNAs, on melanoma progression still requires validation. Notably, since TNC expression in fibroblasts depends on pericellular fibronectin, integrin α5β1 and integrin-linked kinase (ILK-1) [91,92], miRNA regulation of TNC levels in CAFs may occur indirectly via control of these transcription factors involved in TNC expression [93]. Furthermore, TNC can enhance the expression of known melanoma oncomirs like miR-155-5p through a TLR4/NF-kB loop [94].

Laminins are ECM glycoproteins that signal via integrins on melanoma cell surfaces, producing either pro-metastatic [95,96,97] or anti-metastatic outcomes, with let-7 upregulation mediating some of the latter [98]. Depending on the specific laminin subtype or fragment, miRNA-mediated regulation can be oncogenic or antitumoral. While many validated miRNAs regulate laminins and their pathways in various cancers, information specific to skin cancer is scarce. In melanoma, miR-29b has been shown to target laminin gamma 1 (LAMC1), thereby reducing melanoma cell migration and invasion through collagen [99].

For versican, a Wnt pathway effector facilitating cellular adhesion to collagen matrices, there is no miRNA–melanoma-specific information. However, in other cancers, versican is targeted by miR-23b-3p [100], miR-143 [101,102], miR-23b [103] and miR-543 [104], several of which are established melanoma repressors [68,105,106]. Versican can also be upregulated by the actions of miR-5680 [107] and miR-135b [108]—the latter being a validated oncomiR in melanoma [109]. Generally, any oncomiR promoting Wnt signaling could increase versican expression, while tumor suppressor miRNAs inhibiting Wnt may reduce versican transcripts. Versican transcripts act as sponge RNAs modulating other miRNAs and establishing an oncogenic signature seen in hepatocellular carcinoma [110] and in migrating dermal fibroblasts [111], suggesting involvement in melanoma CAFs. Thus, although several miRNAs regulate versican in other malignancies, whether these interactions occur in melanoma remains to be experimentally established (Table 1).

## 4. MicroRNAs Targeting Cell Surface-Associated Extracellular Matrix Molecules

Glypicans contribute to tumorigenesis by modulating various signaling cascades that facilitate tumor progression, including Wnt/β-catenin signaling, bone morphogenic protein (BMP) and fibroblast growth factor (FGF)-mediated pathways. Glypican-6 (GPC6) expression increases in melanoma through HIF-mediated signaling, linked to poor prognosis [112] and correlated with ZEB1 expression and EMT; miR-509-3p directly targets GPC6 [113]. Notably, the promoter for the oncogenic miR-149* (the often-degraded pre-miRNA strand, miR-149-3p) is within the GPC1 gene, and concurrent transcription of miR-149-3p and GPC1 aligns with resistance to apoptosis [114]. In endothelial cells, both strands of miR-149 can regulate GPC1, so the location of miR-149’s promoter within GPC1 indicates intragenic regulation of GPC1 expression [115]—likely impacting neovascularization seen in melanoma. GPC1 positively correlates with melanoma aggressiveness in both melanoma xenograft models [116] and patient samples [117]. Suppressing miR-206, miR-143, or miR-106b is linked to reduced growth and migratory/invasive capacity in multiple melanoma cell lines, mediated by G1 cell cycle arrest and downregulation of CDK4, cyclin D1, cyclin C, and SDC1 or reactivation of p21/WAF1/Cip1 as a target gene [118].

The differential expression of let-7a, characterized by increased levels in melanocytes and decreased or absent expression in melanoma, has been implicated in ECM remodeling. Müller et al. identified let-7a as an inhibitor of *ITGB3* expression, thus suppressing integrin β3 concentrations [119]. As integrin β3 serves as a key mediator of ECM remodeling and is commonly upregulated in melanoma cells, loss of let-7a may significantly influence melanoma-related changes in the TME [120] (Table 1).

## 5. MiRNAs That Indirectly Affect ECM Component Expression

In addition to direct targeting of ECM constituents, numerous miRNAs modulate melanoma progression through indirect regulation of ECM turnover, organization, and signaling [17,18,121,122]. Owing to their ability to simultaneously target multiple transcripts, individual miRNAs exert broad biological effects that extend across interconnected pathways governing proliferation, invasion, angiogenesis, EMT, immune responses, and matrix remodeling. Consequently, many melanoma-associated miRNAs affect ECM homeostasis even when matrix components are not their primary molecular targets. The following section outlines representative oncogenic and tumor-suppressive miRNAs that affect ECM dynamics through the regulation of matrix-remodeling enzymes, signaling intermediates, and transcriptional networks. Although several of the following miRNAs regulate multiple oncogenic pathways, the present discussion focuses specifically on their contributions to ECM remodeling and matrix-dependent melanoma progression.

### 5.1. Oncogenic miRNAs Promoting ECM Remodeling

OncomiRs contribute to tumor development by repressing transcripts implicated in cell cycle arrest and apoptosis or by downregulating tumor suppressor genes and thereby augmenting oncogenic signaling [123]. In the latter scenario, oncomiRs may inhibit negative regulators of oncogenes or activators of tumor suppressor pathways. For example, miR-210 targets MNT, a functional antagonist of MYC, thereby facilitating MYC-driven oncogenic activity [124]. A recent review has catalogued diverse mechanisms by which oncomiRs foster tumorigenesis [125]. For instance, members of the miR-17-92 cluster enhance c-MYC-mediated oncogenic transformation, while miR-372 and miR-373 function independently as oncogenes [126,127,128]. Furthermore, miR-33 and miR-504 directly target p53 mRNA, decreasing p53 protein levels and promoting tumor progression [129]. Many of these miRNAs are also implicated in melanoma progression [130,131,132]. Beyond their intracellular effects, several oncomiRs influence the ECM via paracrine signaling [130,131,132]. For instance, miR-155, part of the miR-17-92-associated regulatory network, can be secreted via exosomes from melanoma cells, facilitating fibroblast differentiation into CAFs, which are major sources of MMP-9 [133,134]. OncomiR expression is mainly orchestrated by key oncogenic transcription factors such as signal transducer and activator of transcription 3 (STAT3), sal-like protein 4 (SALL4), STAT1, and MYC. Otmani et al. have provided a comprehensive overview of miRNAs regulated by these transcription factors [125,135,136,137]. Within melanoma, miR-221/222 are established oncomiRs, targeting c-KIT receptor tyrosine kinase and cyclin-dependent kinase inhibitor p27Kip1 (CDKN1B), thereby promoting proliferation and metastatic potential [138]. While miR-21 is upregulated in primary melanomas, its expression declines in advanced stages, where miR-195 predominates. miR-195 supports melanoma cell proliferation by targeting the WEE1 mitotic inhibitor kinase and promoting migration [139]. Notably, bioinformatic analyses indicate that miR-195-5p may directly target MMP-14, suggesting complex regulatory implications for ECM remodeling [140]. Collectively, these oncomiRs promote melanoma progression not only through intracellular oncogenic signaling but also by enhancing extracellular matrix remodeling, matrix metalloproteinase activity, CAF activation, and the establishment of a pro-invasive tumor microenvironment.

### 5.2. Tumor-Suppressive miRNAs Restricting ECM Remodeling

Tumor-suppressor miRNAs (ts-miRNAs) function to hinder tumor progression by downregulating transcripts encoding oncogenic miRNAs, transcription factors, and proteins involved in cell cycle regulation, proliferation, DNA damage response (DDR), and apoptosis. Numerous ts-miRNA genes are located on chromosome 9p21, a locus frequently deleted in melanoma. Loss of this region results in reduced expression of several tumor-suppressive miRNAs, thereby promoting melanoma progression [141]. Melanoma invasiveness and metastasis are strongly driven by ERK signaling, with miR-876-3p recently identified as a direct ERK inhibitor. However, this miRNA is often lost in melanoma due to its chromosomal location, further linking chromosomal instability to dysregulated signaling cascades [125].

An extensive array of tumor-suppressive miRNAs, including let-7, miR-596, miR-196a, miR-34, miR-193b, miR-532-5p, miR-137, miR-339-3p, miR-204, miR-15a/16-1, and miR-155, has been documented. Although miR-155 is generally considered oncogenic, it may exhibit tumor-suppressive activity depending on tumor stage and cellular environment [142,143].

Due to their capacity to regulate multiple transcripts, ts-miRNAs often coordinate intracellular signaling and ECM remodeling in parallel, impacting metastatic behavior. For instance, miR-204-5p targets both BCL-2 family members and MMP-9, promoting apoptosis while inhibiting invasion and metastasis in melanoma xenograft models. Similarly, the miR-126/126* cluster regulates oncogenic PI3K/c-KIT signaling while also targeting ECM-associated genes, such as ADAM9 and MMP-7 [67].

The let-7 family is among the most thoroughly characterized tumor-suppressive groups, exerting antimetastatic effects in melanoma through the inhibition of integrin-β3 and CDK expression [139]. Let-7 interacts extensively with ECM regulatory networks, influencing and responding to collagen and MMP dynamics, often in a TGF-β-dependent manner [140]. Studies in melanoma and other cancer models demonstrate that let-7 reduces collagen α2 chain expression, as well as MMP-2 and MMP-9 expression, partially via the regulation of basigin and p53 signaling [144,145,146,147,148]. It also suppresses fibronectin, TIMP1, and COL1A1, inhibiting fibroblast-to-myofibroblast differentiation and limiting tumor-supportive stroma formation [149]. Regulation by let-7 is context-dependent; its expression and impact on collagen deposition vary across cancer types and stages, reflecting dynamic ECM remodeling during tumor progression [150]. In melanoma, several let-7 members are downregulated, and miR-107 negatively regulates let-7 in melanoma cell lines [151].

miR-196a is another ts-miRNA frequently reduced in advanced melanoma. It targets the oncogenic transcription factor HOXC8, constraining downstream signaling pathways that drive growth factor production and tumor progression [151]. miR-196a is modulated by TGF-β signaling and engages in ECM regulation, including the control of collagen expression in fibroblasts and other stromal environments [152]. Its function is highly context-dependent, as it can act as an oncogenic miRNA in other cancers by enhancing MMP-2 expression and metastatic properties through annexin 1 targeting [153].

The miR-34 family (miR-34a–c) constitutes a crucial p53-responsive tumor-suppressive network, regulating apoptosis and cell cycle progression via targeting of CDKs and oncogenic transcription factors such as MYC and CREB [154]. In melanoma, miR-34 expression is frequently diminished due to promoter hypermethylation at CpG-rich regions. Beyond intracellular action, miR-34a also modulates ECM remodeling by regulating MMP-2 and MMP-9 expression [155]. Likewise, miR-143 acts as a multi-target tumor suppressor in melanoma, inhibiting MMP-9 and EMT markers, thereby curbing proliferation and promoting apoptosis in vitro [156]. Overall, tumor-suppressive miRNAs restrict melanoma progression by coordinating intracellular tumor-suppressive pathways with the inhibition of ECM remodeling, MMP expression, EMT, and matrix-dependent invasion.

### 5.3. miRNA Regulation of ECM-Associated Transcription Factors

Transcription factor (TF)-mediated gene regulation constitutes a multifaceted and layered system operating alongside miRNA-driven networks. This process involves coordinated alterations in TF expression, activity, and interactions with various upstream and downstream regulatory elements. The intersection of these regulatory pathways affects a wide range of metastasis-associated genes in melanoma. This section provides an overview of key TFs influenced by miRNA-mediated regulation and their essential roles in controlling ECM-related gene expression.

Significant TFs implicated in the development of metastatic melanoma include MITF, AP-1, AP-2, Wnt/β-catenin, NF-κB, TBX3, ETS family members, RUNX2, CREB/ATF-1, ATF2, BPTF, Nrf2, TEAD, HIF, STAT3, SNAIL/SLUG, STAT5, SOX2, BRN2, and PAX3 [157,158,159,160,161,162,163,164]. Transcriptional cofactors such as YAP, TAZ, CRTCs, and enhancer-associated factors like enh17 have also become increasingly prominent [165]. Owing to extensive regulatory interplay, researchers examining miRNA regulation of ECM should also account for TF networks, enhancer and repressor elements, as well as higher-order regulatory mechanisms involving circRNAs and lncRNAs, which collectively influence TF and ECM gene expression outcomes.

Among these TFs, microphthalmia-associated transcription factor (MITF) is recognized as a central lineage-specific oncogenic regulator in melanoma. Tumor progression involves interactions between melanoma cells and adjacent keratinocytes, activating Notch signaling, which leads to MITF dissociation from the miR-221/222 promoter and initiates invasive behavior [166,167]. MITF regulates numerous miRNAs involved in melanoma biology, including miR-125, miR-221/222, let-7, and miR-148b [168,169]. Additionally, MITF influences Dicer transcription, thereby exerting global control over miRNA biogenesis [170]. Functionally, MITF directs phenotypic plasticity in melanoma and modulates ECM-associated genes by decreasing collagen expression and focal adhesion formation, impacting proliferation and migration [171,172] (Table 2).

MITF expression levels closely correlate with invasive behavior: lower MITF is linked to increased MMP-2 expression and greater invasiveness, whereas higher MITF suppresses MT1-MMP and disrupts β-catenin-mediated invasion pathways [173]. The MITF rheostat model describes this dose-dependent regulation, in which different levels of MITF expression are associated with distinct melanoma phenotypes, including proliferation, differentiation, invasion, and therapeutic response [174]. Within this framework, low MITF favors growth arrest and apoptosis, elevated MITF promotes differentiation, and intermediate levels support proliferation. miRNAs are integral to maintaining this equilibrium, contributing to the dynamic expression patterns observed across melanoma stages—primary tumors typically exhibit high MITF, while metastatic lesions tend to display reduced MITF. A reinforcing regulatory loop has been described in which MITF positively regulates miR-579-3p, whereas oncogenic BRAF signaling suppresses MITF expression and consequently reduces miR-579-3p levels, thereby promoting melanoma progression [175].

In early melanoma, several tumor-suppressive miRNAs are unable to regulate MITF due to structural or post-transcriptional obstacles, such as the absence of binding sites within the 3′UTR or masking by RNA-binding proteins. For example, MITF transcripts lack miR-137 binding sites, while miR-340 binding sites may be obscured by CRD-BP [176]. Nevertheless, miRNAs such as miR-137, miR-145, miR-340, miR-101, and miR-148 have been validated to downregulate MITF and suppress melanoma proliferation and invasion [172]. Other miRNAs, including miR-155 and miR-203, indirectly influence MITF through targeting upstream signaling pathways, like Wnt/β-catenin and CREB1 [177].

MITF expression is further regulated by diverse upstream TFs. Positive regulators encompass BRAF-associated signaling pathways, CREB, Wnt/β-catenin, LEF1, SOX10, and ZEB2, whereas negative regulators include TGF-β, BRN2, HOXA1, and DEC1 [178]. Given MITF’s integration within this complex regulatory network, miRNAs targeting any of these upstream TFs can indirectly affect MITF expression and melanoma phenotypes. Furthermore, MITF activity is modified post-translationally via SUMOylation, phosphorylation, and deubiquitination, implying that miRNAs targeting enzymes responsible for these processes can impact MITF function.

Additional pivotal TFs in melanoma progression include CREB1 and AP-2α. AP-2α is generally regarded as a tumor suppressor, with its loss associated with melanoma progression. It stimulates the expression of c-KIT and p53-dependent genes while inhibiting pro-invasive pathways, such as PAR-1 signaling, which governs gap junction proteins, MMP2, and adhesion molecules [158]. AP-2α encourages tumor-suppressive miRNAs such as miR-126/126* while repressing oncogenic miRNAs like miR-25-3p [179,180,181]. During melanoma progression, AP-2α may be directly suppressed by miR-221/222 or indirectly downregulated via miR-214-mediated regulation of AP-2γ [182]. The equilibrium among AP-2 family members, including AP-2ε, contributes to melanoma plasticity and metastatic potential, particularly through the regulation of MMP expression [183].

CREB1 performs multiple functions in melanoma, directly inducing MMP expression, suppressing AP-2α and promoting MITF transcription [184]. It advances proliferation by activating metabolic genes, such as acyl-CoA thioesterase 7 [185,186,187,188], and inhibits RNA-editing enzymes like ADAR1, thus altering miRNA editing and function [166,167]. For instance, unedited miR-378a-3p fails to regulate PARVA, facilitating metastasis. CREB1 also represses ts-miRNAs, including miR-495-5p, and is itself subject to regulation by miRNAs such as miR-203 (direct targeting) and miR-23a-3p (indirect suppression via adenylate cyclase 1) [167,189,190,191].

NF-κB and ETS-1 are major regulators of ECM remodeling in melanoma. NF-κB modulates MMP expression and supports not only tumor growth and invasion but also drug resistance [192,193,194,195]. Its activity can be enhanced by oncogenic miRNAs like miR-21 or inhibited by ts-miRNAs such as miR-377. ETS-1 regulates ECM constituents, including fibronectin, and can also impact NF-κB signaling. It promotes invasion partly by repressing miR-16, which targets the oncogenic factor SOX4 [196]. ETS proteins undergo extensive post-translational modifications, including phosphorylation, acetylation, SUMOylation, and ubiquitination, that influence their stability and activity [197]. Notably, phosphorylation of ETS-1 stabilizes the protein and interferes with miRNA-regulated feedback loops, such as those involving miR-222, thereby sustaining its pro-invasive functions in melanoma [179].

## 6. Clinical Translation, Future Perspectives and Outstanding Challenges

The growing understanding of matrix-miRNAs has generated considerable interest in their potential clinical applications in melanoma. Beyond their mechanistic role in regulating ECM remodeling, these miRNAs represent promising candidates for biomarker development and targeted therapeutic intervention [198,199,200]. However, several biological and technical challenges must be addressed before these findings can be translated into clinical practice.

One of the most promising applications of matrix-miRNAs lies in their use as minimally invasive biomarkers. Circulating miRNAs can be detected in plasma, serum, extracellular vesicles, and other body fluids, where they exhibit remarkable stability due to encapsulation within exosomes or association with RNA-binding proteins [201,202,203]. Several melanoma-associated miRNAs have demonstrated diagnostic and prognostic value, correlating with tumor burden, metastatic progression, patient survival, and therapeutic response [204]. Because ECM remodeling is a hallmark of melanoma invasion and metastasis, circulating matrix-miRNAs may provide dynamic information regarding alterations in the tumor microenvironment, complementing current molecular and histopathological biomarkers.

The therapeutic potential of miRNAs has also attracted increasing attention. Depending on their biological function, tumor-suppressive miRNAs may be restored using synthetic miRNA mimics, whereas oncogenic miRNAs can be inhibited using antisense oligonucleotides, antagomiRs, locked nucleic acids, or miRNA sponges [198,199]. Targeting matrix-miRNAs offers the possibility of simultaneously regulating multiple components of ECM remodeling, including collagen synthesis, matrix metalloproteinases, integrin signaling, angiogenesis, immune regulation, and metastatic dissemination. Such pleiotropic regulation may overcome limitations associated with therapies directed against single molecular targets and may enhance the efficacy of immune checkpoint inhibitors or targeted therapies by remodeling the tumor microenvironment [198,199,205].

Despite these encouraging prospects, several obstacles remain before miRNA-based therapies become clinically feasible. Efficient and tumor-specific delivery remains one of the greatest challenges. Systemically administered miRNAs are susceptible to rapid degradation, renal clearance, immune activation, and unintended uptake by healthy tissues, increasing the risk of off-target effects [198,199]. Lipid nanoparticles, polymeric nanoparticles, viral vectors, engineered exosomes, and ligand-directed delivery systems are currently under intensive investigation to improve stability, tissue specificity, and intracellular delivery [198,199,203]. Nevertheless, ensuring selective accumulation within melanoma cells and stromal compartments while minimizing systemic toxicity remains a major challenge.

Another important consideration is the complexity of miRNA-mediated gene regulation. Individual miRNAs often regulate numerous target genes across multiple signaling pathways, while single ECM-associated genes may be regulated by several distinct miRNAs [206]. Consequently, therapeutic modulation of one miRNA may produce widespread biological effects beyond ECM remodeling. Systems biology approaches integrating transcriptomics, proteomics, epigenomics, and spatial multi-omics technologies will therefore be essential for identifying clinically actionable ECM-miRNA regulatory networks [203,205]. Overall, matrix-miRNAs represent an emerging regulatory interface linking melanoma cells with their extracellular microenvironment. Continued advances in functional validation, biomarker standardization, targeted delivery technologies, and multi-omics analyses will determine whether modulation of ECM-miRNA regulatory networks can be successfully translated into precision medicine approaches for melanoma patients [198,199,202,206].

## 7. Conclusions

A growing body of evidence highlights the pivotal role of miRNAs in regulating ECM remodeling during melanoma progression. By directly targeting matrix components and indirectly influencing signaling pathways, transcription factors, and matrix-remodeling enzymes, miRNAs affect nearly all stages of melanoma progression. These mechanisms are involved in tumor growth, invasion, metastasis, angiogenesis, immune modulation, and therapy resistance. The emerging concept of miRNA–ECM regulatory networks provides a valuable framework for understanding the post-transcriptional mechanisms that shape the melanoma microenvironment.

Unlike the broader landscape of melanoma-associated miRNAs, matrix-miRs specifically regulate ECM composition, remodeling, and cell–matrix communication, thereby influencing both the tumor cells and their surrounding microenvironment. Although substantial progress has been made in identifying these regulatory networks, many proposed miRNA–ECM interactions remain to be experimentally validated in melanoma, particularly those extrapolated from other cancer models. Future studies integrating functional validation with multi-omics approaches will be essential to define clinically relevant matrix-miRNA networks and facilitate their translation into biomarkers and therapeutic strategies for precision medicine. Ultimately, validation of matrix-regulating miRNAs as biomarkers and therapeutic targets may enable the development of novel treatment strategies that simultaneously target melanoma cells and the supportive microenvironment, thereby improving disease progression and patient outcomes.

## Figures and Tables

**Table 1:** MicroRNAs regulating ECM-associated molecules implicated in melanoma progression. The table summarizes validated or predicted miRNA–target interactions, their reported effects on melanoma biology, functional outcomes, and the level of supporting evidence.

ECM Target	miRNA(s)	Direct Target	Mode of ECM Regulation	Effect in Melanoma	Functional Outcome	Evidence
CTHRC1	miR-134	✓	Direct ECM target	Tumor suppressor	↓ Collagen synthesis, ↓ invasion, ↓ metastasis	Melanoma
LOX	miR-30a	✓	ECM-remodeling enzyme	Tumor suppressor	↓ Collagen cross-linking, ↓ metastasis	Melanoma
POSTN	miR-214	✓	Direct ECM target	Tumor suppressor	↓ EMT, ↓ invasion	Melanoma
PHs	miR-124, miR-429	✓	ECM-remodeling enzyme	Tumor suppressor	↓ Collagen cross-linking	Melanoma
CD44	miR-199a-5p, miR-143-3p, miR-328	✓	ECM-associated receptor	Tumor suppressor	↓ Migration, ↓ proliferation	Melanoma/ other cancers
CEMIP	miR-140-5p, miR-296-3p, miR-216a-5p	✓	ECM-remodeling enzyme	Tumor suppressor	↓ Hyaluronan degradation	Melanoma
HAS isoforms	miR-200c, miR-296-3p, miR-376, miR-10a-5p, miR-29, miR-125a	✓	ECM biosynthesis enzymes	Suppression	↓ Hyaluronan synthesis	Melanoma
FΝ1	miR-509-3p	✓	Direct ECM target	Tumor suppressor	↓ Migration	Melanoma
LAMC1	miR-29b	✓	ECM basement membrane	Tumor suppressor	↓ Migration, ↓ invasion	Melanoma
VCAN	miR-23b-3p, miR-143, miR-543, miR-135b	✓/ Predicted *	Direct ECM target	Controversial	Wnt signaling, migration	Other cancers (extrapolated) *
GPC6	miR-509-3p	✓	Cell surface- associated ECM	Tumor suppressor	↓ EMT	Melanoma
GPC1	miR-149	✓	Cell surface- associated ECM	Context- dependent role	Angiogenesis/apoptosis	Melanoma/ xenograft models
ITGB3	let-7a	✓	ECM receptor	Tumor suppressor	↓ ECM remodeling	Melanoma

* Predicted interactions indicate bioinformatic predictions without direct experimental validation in melanoma. Evidence classified as “Other cancers (extrapolated)” refers to findings reported in non-melanoma tumor models that remain to be validated in melanoma.

**Table 2:** miRNA–transcription factor regulatory networks controlling extracellular matrix (ECM) remodeling in melanoma. The table summarizes representative miRNA-mediated regulation of transcription factors that indirectly influence ECM-associated genes and pathways involved in melanoma progression. The corresponding regulatory mechanisms and the level of supporting evidence, including melanoma-specific and extrapolated findings where applicable, are discussed in detail in the main text.

ECM Molecules Affected by miRNAs	Targeted Transcription Factor	miRNAs
Collagen	MITF	miR-137, miR-145, miR-340, miR-101, miR-148
Focal adhesions	MITF	miR-137, miR-145, miR-340, miR-101, miR-148
MMP-2	MITF, AP-2α	miR-137, miR-145, miR-340, miR-101, miR-148, miR-221/222, miR-214
MT1-MMP	MITF	miR-137, miR-145, miR-340, miR-101, miR-148, miR-579-3p
MMPs	CREB1, NF-κB	miR-203, miR-23a-3p, miR-495-5p, miR-21, miR-377
FN1	ETS-1	miR-16, miR-222
Adhesion molecules/ gap junction proteins	AP-2α	miR-221/222, miR-214
PARVA	CREB1–ADAR1 axis	miR-378a-3p

## Data Availability

The original contributions presented in this study are included in the article. Further inquiries can be directed to the corresponding author.

## Abbreviations

CAFs	Cancer-associated fibroblasts
ECM	Extracellular matrix
EMT	Epithelial–mesenchymal transition
GAGs	Glycosaminoglycans
HAS	Hyaluronan synthase
HAPLN1	Hyaluronan and proteoglycan link protein 1
HSPGs	Heparan sulfate proteoglycans
ILK	Integrin-linked kinase
LOX	Lysyl oxidase
LOXL	Lysyl oxidase-like enzyme
MMP	Matrix metalloproteinase
miRNA	MicroRNA
MITF	Microphthalmia-associated transcription factor
NF-κB	Nuclear factor kappa-light-chain-enhancer of activated B cells
PGs	Proteoglycans
POSTN	Periostin
SDC	Syndecan
TME	Tumor microenvironment
TNC	Tenascin-C
TF	Transcription factor
WISP	Wnt1-inducible signaling pathway protein
